# Supplementary material for: HIVprotI: an integrated web based platform for prediction and design of HIV proteins inhibitors
Source: J Cheminform. 2018 Mar 9;10:12. doi: 10.1186/s13321-018-0266-y (PMC5845081; doi:10.1186/s13321-018-0266-y)
Supplement: Supplementary file 2 — Additional file 2. Source code of HIVProtI web server. [file 13321_2018_266_MOESM2_ESM.zip › HIVprotI_Source-code/hivprotI/weka-3-6-6/doc/weka/classifiers/lazy/LBR.Indexes.html]

LBR.Indexes


---


|  |  |  |
| --- | --- | --- |
| **Overview** \| **Package** \| **Class** \| **Tree** \| **Deprecated** \| **Index** \| **Help** \| **Weka's home** | |  |
| **PREV CLASS**   **NEXT CLASS** | **FRAMES**    **NO FRAMES**     **All Classes** |  |
| SUMMARY: NESTED \| FIELD \| CONSTR \| METHOD | DETAIL: FIELD \| CONSTR \| METHOD |  |


---


## weka.classifiers.lazy Class LBR.Indexes

```
java.lang.Object
  weka.classifiers.lazy.LBR.Indexes
```

**All Implemented Interfaces:**: java.io.Serializable, RevisionHandler

**Enclosing class:**: LBR

---

``` public class LBR.Indexes extends java.lang.Object implements java.io.Serializable, RevisionHandler ```

Class for handling instances and the associated attributes.

Enables a set of indexes to a given dataset to be created and used
with an algorithm. This reduces the memory overheads and time required
when manipulating and referencing Instances and their Attributes.

**See Also:**: Serialized Form

---

| **Field Summary** | |
| --- | --- |
| `boolean[]` | `m_AttIndexes`             the array attribute indexes |
| `int` | `m_ClassIndex`             the Class Index for the data set |
| `boolean[]` | `m_InstIndexes`             the array instance indexes |
| `int` | `m_NumAttsSet`             the number of attributes "in use" or set to a the original value (true or false) |
| `int` | `m_NumInstsSet`             the number of instances "in use" or set to a the original value (true or false) |
| `int` | `m_NumSeqAttsSet`             the number of sequential attributes "in use" or set to a the original value (true or false) |
| `int` | `m_NumSeqInstsSet`             the number of sequential instances "in use" or set to a the original value (true or false) |
| `int[]` | `m_SequentialAttIndexes`             an array of attribute indexes that are set to either true or false |
| `int[]` | `m_SequentialInstIndexes`             the array of instance indexes that are set to a either true or false |


**Constructor Summary**

`LBR.Indexes(int numInstances,
int numAtts,
boolean value,
int classIndex)`
  
          constructor

`LBR.Indexes(LBR.Indexes FromIndexes)`
  
          constructor


| **Method Summary** | |
| --- | --- |
| `boolean` | `getAttIndex(int index)`             Returns the boolean value at the specified index in the Attribute Indexes array |
| `boolean` | `getInstanceIndex(int index)`             Returns the boolean value at the specified index in the Instance Index array |
| `int` | `getNumAttributes()`             Returns the number of attributes in the dataset |
| `int` | `getNumAttributesSet()`             Returns the number of attributes "in use" |
| `int` | `getNumInstances()`             Returns the number of instances in the dataset |
| `int` | `getNumInstancesSet()`             Returns the number of instances "in use" |
| `java.lang.String` | `getRevision()`             Returns the revision string. |
| `int` | `getSequentialAttIndex(int index)`             Returns the boolean value at the specified index in the Sequential Attribute Indexes array |
| `int` | `getSequentialInstanceIndex(int index)`             Returns the boolean value at the specified index in the Sequential Instance Indexes array |
| `int` | `getSequentialNumAttributes()`             Returns the number of attributes in the Sequential array |
| `int` | `getSequentialNumInstances()`             Returns the number of instances in the Sequential array |
| `boolean` | `isSequentialAttIndexValid()`             Returns whether or not the Sequential Attribute Index requires rebuilding due to a change |
| `boolean` | `isSequentialInstanceIndexValid()`             Returns whether or not the Sequential Instance Index requires rebuilding due to a change |
| `void` | `resetAttIndex(boolean value)`             Resets the boolean value in AttIndexes array |
| `void` | `resetAttIndexTo(LBR.Indexes FromIndexes)`             Resets the boolean value in AttIndexes array based on another set of Indexes |
| `void` | `resetDatasetBasedOn(LBR.Indexes FromIndexes)`             Resets the boolean values in Attribute and Instance array to reflect an empty dataset withthe same attributes set as in the incoming Indexes Object |
| `void` | `resetInstanceIndex(boolean value)`             Resets the boolean value in the Instance Indexes array to a specified value |
| `void` | `setAttIndex(int index, boolean value)`             Changes the boolean value at the specified index in the AttIndexes array |
| `void` | `setAtts(int[] Attributes, boolean value)`             Changes the boolean value at the specified index in the InstIndexes array |
| `void` | `setInstanceIndex(int index, boolean value)`             Changes the boolean value at the specified index in the InstIndexes array |
| `void` | `setInsts(int[] Instances, boolean value)`             Changes the boolean value at the specified index in the InstIndexes array |
| `void` | `setSequentialAttIndex(boolean value)`             A Sequential Attribute index is all those Attributes that are set to the specified value placed in a sequential array. |
| `void` | `setSequentialDataset(boolean value)`             Sets both the Instance and Attribute indexes to a specified value |
| `void` | `setSequentialInstanceIndex(boolean value)`             A Sequential Instance index is all those Instances that are set to the specified value placed in a sequential array. |

**Methods inherited from class java.lang.Object**

`equals, getClass, hashCode, notify, notifyAll, toString, wait, wait, wait`

**Field Detail**

### m\_InstIndexes

```
public boolean[] m_InstIndexes
```

:   the array instance indexes

---


### m\_AttIndexes

```
public boolean[] m_AttIndexes
```

:   the array attribute indexes

---


### m\_SequentialInstIndexes

```
public int[] m_SequentialInstIndexes
```

:   the array of instance indexes that are set to a either true or false

---


### m\_SequentialAttIndexes

```
public int[] m_SequentialAttIndexes
```

:   an array of attribute indexes that are set to either true or false

---


### m\_NumInstsSet

```
public int m_NumInstsSet
```

:   the number of instances "in use" or set to a the original value (true or false)

---


### m\_NumAttsSet

```
public int m_NumAttsSet
```

:   the number of attributes "in use" or set to a the original value (true or false)

---


### m\_NumSeqInstsSet

```
public int m_NumSeqInstsSet
```

:   the number of sequential instances "in use" or set to a the original value (true or false)

---


### m\_NumSeqAttsSet

```
public int m_NumSeqAttsSet
```

:   the number of sequential attributes "in use" or set to a the original value (true or false)

---


### m\_ClassIndex

```
public int m_ClassIndex
```

:   the Class Index for the data set


**Constructor Detail**

### LBR.Indexes

```
public LBR.Indexes(int numInstances,
                   int numAtts,
                   boolean value,
                   int classIndex)
```

:   constructor

    **Parameters:**: `numInstances` - the number of instances in dataset: `numAtts` - the number of attributes in dataset: `value` - either true or false: `classIndex` - Set to -1 if you want class attribute switched on or the value of the instances class index will be switched of and the class attibute will not be considered.

---


### LBR.Indexes

```
public LBR.Indexes(LBR.Indexes FromIndexes)
```

:   constructor

    **Parameters:**: `FromIndexes` - the object you want to copy


**Method Detail**

### setInstanceIndex

```
public void setInstanceIndex(int index,
                             boolean value)
```

:   Changes the boolean value at the specified index in the InstIndexes array

    :   **Parameters:**: `index` - the index of the instance: `value` - the value to set at the specified index

---


### setAtts

```
public void setAtts(int[] Attributes,
                    boolean value)
```

:   Changes the boolean value at the specified index in the InstIndexes array

    :   **Parameters:**: `Attributes` - array of attributes: `value` - the value to set at the specified index

---


### setInsts

```
public void setInsts(int[] Instances,
                     boolean value)
```

:   Changes the boolean value at the specified index in the InstIndexes array

    :   **Parameters:**: `Instances` - array of instances: `value` - the value to set at the specified index

---


### setAttIndex

```
public void setAttIndex(int index,
                        boolean value)
```

:   Changes the boolean value at the specified index in the AttIndexes array

    :   **Parameters:**: `index` - the index of the instance: `value` - the value to set at the specified index

---


### getInstanceIndex

```
public boolean getInstanceIndex(int index)
```

:   Returns the boolean value at the specified index in the Instance Index array

    :   **Parameters:**: `index` - the index of the instance **Returns:**: the boolean value at the specified index

---


### getSequentialInstanceIndex

```
public int getSequentialInstanceIndex(int index)
```

:   Returns the boolean value at the specified index in the Sequential Instance Indexes array

    :   **Parameters:**: `index` - the index of the instance **Returns:**: the requested value

---


### resetInstanceIndex

```
public void resetInstanceIndex(boolean value)
```

:   Resets the boolean value in the Instance Indexes array to a specified value

    :   **Parameters:**: `value` - the value to set all indexes

---


### resetDatasetBasedOn

```
public void resetDatasetBasedOn(LBR.Indexes FromIndexes)
```

:   Resets the boolean values in Attribute and Instance array to reflect an empty dataset withthe same attributes set as in the incoming Indexes Object

    :   **Parameters:**: `FromIndexes` - the Indexes to be copied

---


### resetAttIndex

```
public void resetAttIndex(boolean value)
```

:   Resets the boolean value in AttIndexes array

    :   **Parameters:**: `value` - the value to set the attributes to

---


### resetAttIndexTo

```
public void resetAttIndexTo(LBR.Indexes FromIndexes)
```

:   Resets the boolean value in AttIndexes array based on another set of Indexes

    :   **Parameters:**: `FromIndexes` - the Indexes to be copied

---


### getAttIndex

```
public boolean getAttIndex(int index)
```

:   Returns the boolean value at the specified index in the Attribute Indexes array

    :   **Parameters:**: `index` - the index of the Instance **Returns:**: the boolean value

---


### getSequentialAttIndex

```
public int getSequentialAttIndex(int index)
```

:   Returns the boolean value at the specified index in the Sequential Attribute Indexes array

    :   **Parameters:**: `index` - the index of the Attribute **Returns:**: the requested value

---


### getNumInstancesSet

```
public int getNumInstancesSet()
```

:   Returns the number of instances "in use"

    :   **Returns:**: the number of instances "in use"

---


### getNumInstances

```
public int getNumInstances()
```

:   Returns the number of instances in the dataset

    :   **Returns:**: the number of instances in the dataset

---


### getSequentialNumInstances

```
public int getSequentialNumInstances()
```

:   Returns the number of instances in the Sequential array

    :   **Returns:**: the number of instances in the sequential array

---


### getNumAttributes

```
public int getNumAttributes()
```

:   Returns the number of attributes in the dataset

    :   **Returns:**: the number of attributes

---


### getNumAttributesSet

```
public int getNumAttributesSet()
```

:   Returns the number of attributes "in use"

    :   **Returns:**: the number of attributes "in use"

---


### getSequentialNumAttributes

```
public int getSequentialNumAttributes()
```

:   Returns the number of attributes in the Sequential array

    :   **Returns:**: the number of attributes in the sequentual array

---


### isSequentialInstanceIndexValid

```
public boolean isSequentialInstanceIndexValid()
```

:   Returns whether or not the Sequential Instance Index requires rebuilding due to a change

    :   **Returns:**: true if the sequential instance index needs rebuilding

---


### isSequentialAttIndexValid

```
public boolean isSequentialAttIndexValid()
```

:   Returns whether or not the Sequential Attribute Index requires rebuilding due to a change

    :   **Returns:**: true if the sequential attribute index needs rebuilding

---


### setSequentialDataset

```
public void setSequentialDataset(boolean value)
```

:   Sets both the Instance and Attribute indexes to a specified value

    :   **Parameters:**: `value` - the value for the Instance and Attribute indices

---


### setSequentialInstanceIndex

```
public void setSequentialInstanceIndex(boolean value)
```

:   A Sequential Instance index is all those Instances that are set to the specified value placed in a sequential array.
    Each value in the sequential array contains the Instance index within the Indexes.

    :   **Parameters:**: `value` - the sequential instance index

---


### setSequentialAttIndex

```
public void setSequentialAttIndex(boolean value)
```

:   A Sequential Attribute index is all those Attributes that are set to the specified value placed in a sequential array.
    Each value in the sequential array contains the Attribute index within the Indexes

    :   **Parameters:**: `value` - the sequential attribute index

---


### getRevision

```
public java.lang.String getRevision()
```

:   Returns the revision string.

    :   **Specified by:**: `getRevision` in interface `RevisionHandler`
    :   **Returns:**: the revision


---


|  |  |  |
| --- | --- | --- |
| **Overview** \| **Package** \| **Class** \| **Tree** \| **Deprecated** \| **Index** \| **Help** \| **Weka's home** | |  |
| **PREV CLASS**   **NEXT CLASS** | **FRAMES**    **NO FRAMES**     **All Classes** |  |
| SUMMARY: NESTED \| FIELD \| CONSTR \| METHOD | DETAIL: FIELD \| CONSTR \| METHOD |  |


---
